# LDHB silencing enhances the effects of radiotherapy by impairing nucleotide metabolism and promoting persistent DNA damage

**DOI:** 10.1038/s41598-025-95633-3

**Published:** 2025-03-29

**Authors:** Haibin Deng, Fatlind Malsiu, Huixiang Ge, Tereza Losmanova, Michaela Medová, Nicola Zamboni, Wenxiang Wang, Ren-Wang Peng, Jinming Tang, Patrick Dorn, Thomas Michael Marti

**Affiliations:** 1 Department of General Thoracic Surgery, Inselspital Bern University Hospital, Murtenstrasse 28, 3008 Bern, Switzerland; 2 Department of BioMedical Research, University of Bern, Bern, Switzerland; 3 Graduate School of Cellular and Biomedical Sciences, University of Bern, Bern, Switzerland; 4 Institute of Tissue Medicine and Pathology, University of Bern, Bern, Switzerland; 5 Department of Radiation Oncology, Inselspital, Bern University Hospital, Bern, Switzerland; 6 Institute for Molecular Systems Biology, ETH Zurich, Zurich, Switzerland; 7 2nd Department of Thoracic Surgery, The Affiliated Cancer Hospital of Xiangya School of Medicine, Central South University/Hunan Cancer Hospital Hunan, 582 Xianjiahu Rd, Yuelu, Changsha, 410013 Hunan China

**Keywords:** Lung cancer, Lactate dehydrogenase, Nucleotide metabolism, DNA damage, Radiotherapy, Cancer therapy, Radiotherapy, Cancer metabolism

## Abstract

Lung cancer is the leading cause of cancer-related deaths globally, with radiotherapy as a key treatment modality for inoperable cases. Lactate, once considered a by-product of anaerobic cellular metabolism, is now considered critical for cancer progression. Lactate dehydrogenase B (LDHB) converts lactate to pyruvate and supports mitochondrial metabolism. In this study, a re-analysis of our previous transcriptomic data revealed that LDHB silencing in the NSCLC cell lines A549 and H358 dysregulated 1789 genes, including gene sets associated with cell cycle and DNA repair pathways. LDHB silencing increased H2AX phosphorylation, a surrogate marker of DNA damage, and induced cell cycle arrest at the G1/S or G2/M checkpoint depending on the p53 status. Long-term LDHB silencing sensitized A549 cells to radiotherapy, resulting in increased DNA damage and genomic instability as evidenced by increased H2AX phosphorylation levels and micronuclei accumulation, respectively. The combination of LDHB silencing and radiotherapy increased protein levels of the senescence marker p21, accompanied by increased phosphorylation of Chk2, suggesting persistent DNA damage. Metabolomics analysis revealed that LDHB silencing decreased nucleotide metabolism, particularly purine and pyrimidine biosynthesis, in tumor xenografts. Nucleotide supplementation partially attenuated DNA damage caused by combined LDHB silencing and radiotherapy. These findings suggest that LDHB supports metabolic homeostasis and DNA damage repair in NSCLC, while its silencing enhances the effects of radiotherapy by impairing nucleotide metabolism and promoting persistent DNA damage.

## Introduction

Lung cancer is the leading cause of cancer-related deaths worldwide, with more than 80% of lung tumors being non-small cell lung cancer (NSCLC) (reviewed in^1^). Radiotherapy (RT) was established as a treatment option for medically inoperable or borderline operable patients. However, 5 years after treatment with surgery or elective RT, operable patients suffer from local and distal relapse in approximately 20% and 34% of all cases, respectively^2^.

Although lactate is generally considered a waste product of anaerobic glycolysis, e.g., the Warburg effect, lactate has been shown to serve as the primary carbon source for the TCA cycle in vivo, providing substrate and electrons for oxidative phosphorylation in both normal tissue and lung tumors^3,4^. The interconversion of pyruvate and lactate using NADH/NAD^+^ as a co-substrate is catalyzed by the tetrameric enzyme lactate dehydrogenase (LDH), which is encoded by the genes lactate dehydrogenase A and B (*LDHA* and *LDHB*, respectively) (reviewed in^5^). LDHA, particularly the LDHA-homo-tetramer, converts pyruvate to lactate, whereas LDHB primarily converts lactate to pyruvate. LDHB is localized in the mitochondria; it is ubiquitously expressed and is the predominant isoform in cardiac muscle^6^. Only the double-knockout of *LDHA* and *LDHB* entirely suppressed LDH activity and lactate secretion^7^. Thus, LDHB may at least partially substitute for the function of LDHA in promoting glycolysis.

The relationship between LDHB expression and cancer is complex: LDHB is silenced by promoter methylation in several cancers, but increased LDHB expression has been described in several adenocarcinomas, including NSCLC (see^8^ for references). LDHB expression correlates with response to neoadjuvant chemotherapy in breast cancer^9^. However, to the best of our knowledge, there is only one report in the literature describing how targeting LDHB affects cancer therapy response. In detail, it was shown that silencing of LDHB renders oral squamous cell carcinoma cells more sensitive to Taxol^10^, which is a radiosensitizer. Interesting in the context of this study, the inducible inhibition of LDHA significantly increased the sensitivity of head and neck xenograft tumors to radiation therapy^11^. However, to the best of our knowledge, there are no studies on how silencing LDHB affects the response of cancer cells to radiotherapy. Thus, the aim of this study is to elucidate how LDHB silencing affects DNA damage levels in cancer cells, either by itself or when combined with radiotherapy.

## Methods

### Cell culture

All cell lines were obtained from ATCC (American Type Culture Collection), except the patient-derived primary LUAD cells PF139 that were established as recently reported^12^. Cells were cultured in the RPMI medium (8758; Sigma-Aldrich 8758) or DMEM/F12 (Life Technologies 21,331,020) containing 2mM L-glutamine (Life Technologies 25,030,024), 10% fetal bovine serum/FBS (Life Technologies 10,270–106), and 1% penicillin/streptomycin solution (Sigma-Aldrich P0781) at 37 °C in a humidified 5% CO_2_ incubator.

### Flow cytometry

Cells were harvested and fixed with IC fixation buffer (Thermo Fisher Scientific 00–8222-49) for 15 min and then permeabilized with 0.1% Triton X100 (Cat. #X100; Sigma-Aldrich) for 10 min. Subsequently, cells were incubated in 200 μl PBS containing 2% FBS and 0.25% Fc Receptor Binding Inhibitor Functional Grade Monoclonal Antibody for 10 min at room temperature and stained with Alexa Fluor® 488 anti-H2A.X-Phosphorylated (BioLegend 613,406) overnight at 4°C. After washing with 2% FBS, cells were resuspended in 2% FBS containing 0.5 g/mL DAPI. All samples were analyzed using a LSRII flow cytometer, and 10,000 events were recorded.

### Immunoblotting

Cell lysates were extracted in RIPA buffer (Sigma Aldrich Chemie GmbH R0278) with 1 × Protease and Phosphatase (Thermo Scientific 78,444) for 20 min on ice, and protein was harvested by centrifugation at 14,000g for 25 min at 4°C. BCA Protein Assay Kit (Thermo Scientific 23,223) was used for protein quantification. Samples were separated by SDS-PAGE and transferred to Nitrocellulose membrane (Bio-Rad 1,704,158), blocked in TBS (LI-COR Biosciences 927–60,001) for 2 h at room temperature and incubated with PARP (Cell Signaling Technology 9532S, 1:1000), p-Chk2 (Cell Signaling Technology 2197S, 1:1000), P53 (Cell Signaling Technology 9282S, 1:1000), P21 (Abcam ab109520, 1:1000), LDHB (R&D Systems MAB9205-100, 1:20,000) overnight at 4°C. The membranes were then incubated with secondary antibodies (Li-COR Biosciences 926–68,020 and 926–32,211, 1:5000) for 45 min at room temperature. Images were acquired and analyzed using Image Studio.

### Senescence β-Galactosidase Staining

The Senescence β-Galactosidase Staining Kit from Cell Signaling Technology (Cell Signaling Technology 9860S) was used for senescence analysis according to the manufacturer’s protocol. In brief, 50,000 cells were seeded in 6-well plates and irradiated with different doses the following day. After 5 days, the cells were fixed and stained overnight at 37° with β-galactosidase staining solution. The images were taken under the microscope.

### Immunofluorescence and confocal microscopy

20′000 cells were grown on 4-well chamber slides (Thermo Scientific Nunc 154,526) for two days, then fixed with 4% paraformaldehyde for 20 min at RT and permeabilized with 0.2% Triton X-100 for 15 min. The cells were then treated with acetone and methanol (1:1) for 20 min at room temperature. After blocking with 1% BSA for 2 h at room temperature, staining with Alexa Fluor® 647 anti-H2A.X-Phosphorylated (Ser139) Antibody (BioLegend 613,408) overnight at 4°C was performed. Slides were covered with cover slips and mounting buffer containing DAPI (Fisher Scientific P-36931). Images were acquired by ZEISS_LSM 710 confocal microscope and analyzed with Fiji.

### Immunohistochemistry

Immunohistochemistry was performed at room temperature using the fully automated BOND RX® staining system (Leica Biosystems) as previously described^13^. Samples were stained with LDHB (R&D Systems MAB9205-100, 1:5000).

Phospho-Histone H2A.X (Ser139) (20E3) Rabbit mAb (Cell Signaling Technology 9718S, 1:200), p21 Waf1/Cip1 (12D1) Rabbit mAb (Cell Signaling Technology 2947S, 1:400). The images were analyzed with Qupath.

### Gene silencing by small interfering (siRNA) and short hairpin RNAs (shRNA)

For transient silencing, cells were transfected with the mixtures of lipofectamine 2000 (Invitrogen 11,668,027) with pooled LDHB human siRNA Oligo Duplex (OriGene Technologies SR320835) or Universal Scrambled Negative Control siRNA Duplex. For stable knockdown, the lentiviruses were produced from the LDHB Human shRNA Plasmid Kit (Origene TL311768) according to The RNAi Consortium (TRC) Broad Institute protocol. Cells were infected with lentivirus and selected with 2 μg/mL puromycin for three days. Colonies were isolated using 8 mm cloning cylinders (MERCK Millipore TR-1004) and the knockdown was validated by Western blot.

### LC–MS

LC–MS experiments were performed as described previously^14^. In brief, 5 × 10^5^ cells transfected with siRNAs were culture in 6-well plates medium for 48 h. Then, cells washed with 2 mL of prewarmed (37 °C) wash solvent (75 mM ammonium carbonate, pH was adjusted to 7.4 with acetic acid) and extracted with 400 μl of the pre-cooled (-20 °C) extraction solvent (40% acetonitrile, 40% methanol, 20% nanopure water). The metabolites were extracted by scraping on ice, and then clean supernatants were harvested after spinning at 5000 g for 5 min at 4 °C. The samples were immediately stored at -80 °C for further analysis by LC–MS measurements, and the data was analyzed as described previously^15^.

### Animal experiments

The animal experiments were performed in accordance with animal welfare guidelines and protocols approved by the ethical committee of the canton of Bern, Switzerland (license number BE49/2022. The study was reported in accordance with ARRIVE guidelines (https://arriveguidelines.org). For tumor inoculation, 1 × 10^6^ shCTRL or shLDHB A549 cells suspended in 100 μl PBS and growth factor-reduced Matrigel (1:1) were injected subcutaneously (left and right flank) into RAG mice. When tumor volume reached 40–100 mm^3^, the mice were randomly assigned to different treatment groups and then were irradiated locally using an X-RAD SmART irradiator (Precision X-Ray) with a single dose of 10 Gy.

### Public data source and analysis

The single-cell RNA sequencing data was collected and analyzed in the online tool CancerSEA (http://biocc.hrbmu.edu.cn/CancerSEA/) using expression data from Guillaumet-Adkins A. Genome Biol. 2017 (PDX_LUAD) and Lambrechts D. Nat Med. 2018 (Lung)^16^ . The genes positively correlated with LDHB were generated by cBioPortal (https://www.cbioportal.org/) using the expression data from Cancer Cell Line Encyclopedia (Broad, 2019). The genes were then used for enrichment analysis by using Enrichr (https://maayanlab.cloud/Enrichr/enrich) and visualized by Appyter (https://appyters.maayanlab.cloud/#/)^17^.

### Statistical analysis

Statistical analysis was performed with GraphPad Prism 9. Repeated measurements were performed on different biological samples. Pearson R was used for the correlation analysis. The statistical significance of the correlation was determined by a two-tailed t-test. The error bars represent the mean ± standard deviation (SD). Two-tailed unpaired, unpaired Welch’s t-test, paired Student’s t-test was performed as described in the figure legends. The p-values < 0.05 were considered significant. In all analyses, the significance level is indicated as follows: *P < 0.05, **P < 0.01, ***P < 0.001, ****P < 0.0001*.

## Results

### LDHB expression is linked to the activation of DNA damage response and its silencing increases DNA damage accumulation

**Fig.1:**
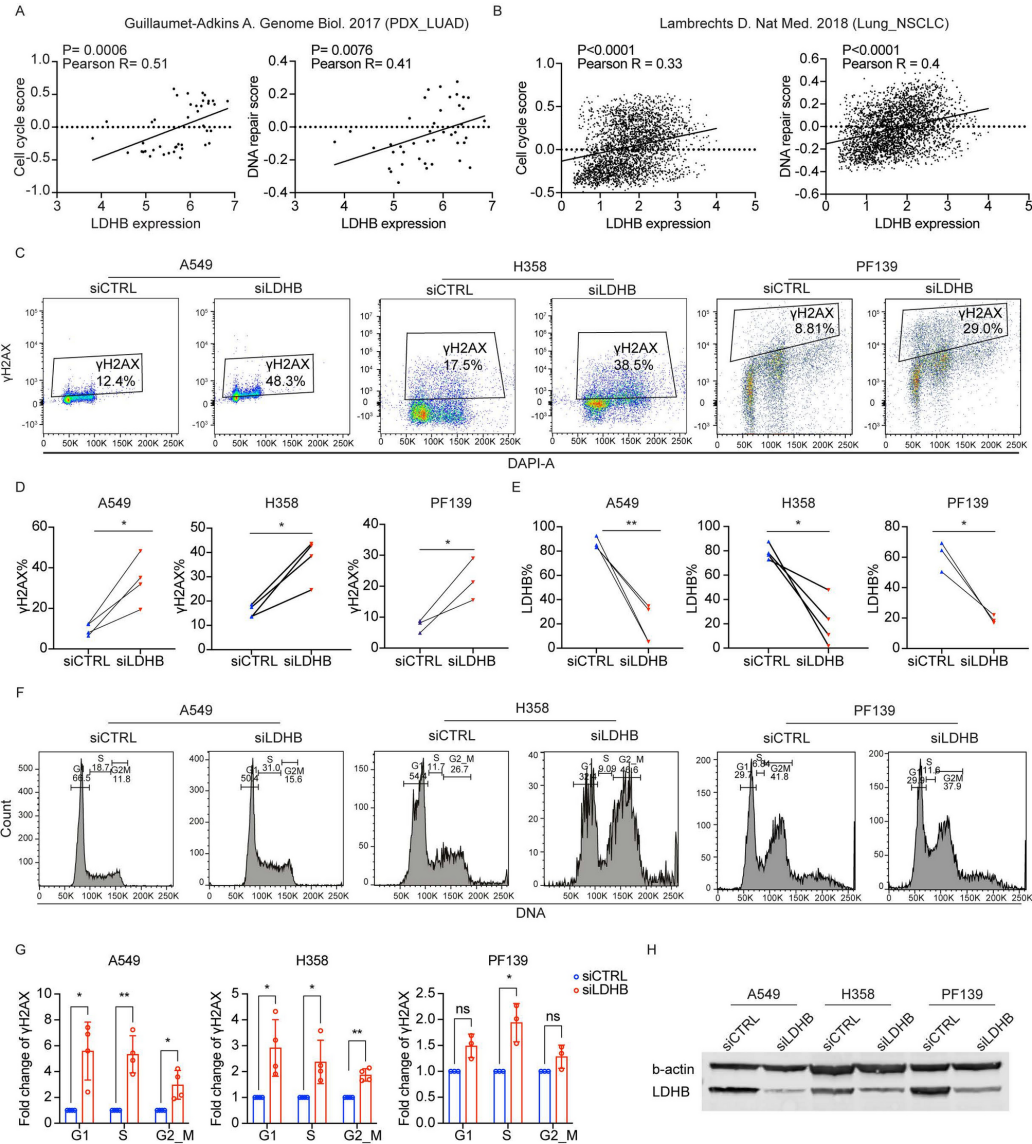
LDHB expression is linked to the activation of DNA damage response and its silencing increases DNA damage accumulation. **A**-**B**, correlation analysis of LDHB expression with cell cycle and DNA repair score was performed 3 using CancerSEA. **C**-**E**, the protein expression of LDHB and γH2AX was analyzed by flow cytometry 72 h after 4 transfection with siCTRL or siLDHB (n = 3–4), and significance was evaluated using paired T-test. **F**, the cell cycle 5 distribution was analyzed 72 h after transfection with siCTRL or siLDHB by flow cytometry using DAPI staining. **G**, 6 the γH2AX expression were analysis by flow cytometry at different cell cycle (n = 3–4). data were means ± SD, unpaired 7 Welch’s t-test. **H**, LDHB expression in A549, H358, PF139-siCTRL and siLDHB cells was analyzed by Western blot after 8 72 h of transfection. 9

Our previously performed comprehensive whole transcriptome expression analysis revealed that LDHB silencing reduced the expression of 1789 genes in common between the A549 and the H358 cell lines^18^. Pathway enrichment analysis revealed that among the top 25 most downregulated gene sets, eight pathways were related to cell proliferation and cell cycle progression^18^. In this study, our in silico Gene Set Enrichment Analysis of data available from the CancerSEA revealed that LDHB expression positively correlated with a “Cell cycle score” and a “DNA repair score” in patient-derived xenograft samples and also in primary NSCLC samples at single-cell resolution (Fig. 1A-B). Furthermore, increased LDHB expression in lung adenocarcinomas was significantly correlated with enriched expression of genes associated with the DNA damage response, such as DNA repair, cell cycle, and purine/pyrimidine biosynthesis (Fig. S1A-D). LDHB silencing in NSCLC cell lines A549, H358, and primary culture PF139^19^ significantly increased H2AX phosphorylation, a marker of DNA damage^20^, compared to controls (Fig. 1C-E, H, Fig. S1E). Silencing LDHB in p53-proficient A549 cells caused a cell cycle arrest in the S-phase (Fig. 1F, left panels). Similarly, after LDHB silencing, most PF139 cells also accumulated in the G1/S-phase, suggesting that these cells are p53-proficient^21^ (Fig. 1F, right panels). In contrast, p53-deficient H358 cells showed a marked accumulation in the G2/M phase (Fig. 1F, middle panels). In addition, H2AX phosphorylation was significantly increased in S phase in all cell lines tested, and additionally in G1 and G2/M phases in A549 and H358 cells after LDHB silencing (Fig. 1G, Fig. S1F).

### Long-term LDHB silencing renders cells sensitive to radiotherapy

**Fig.2:**
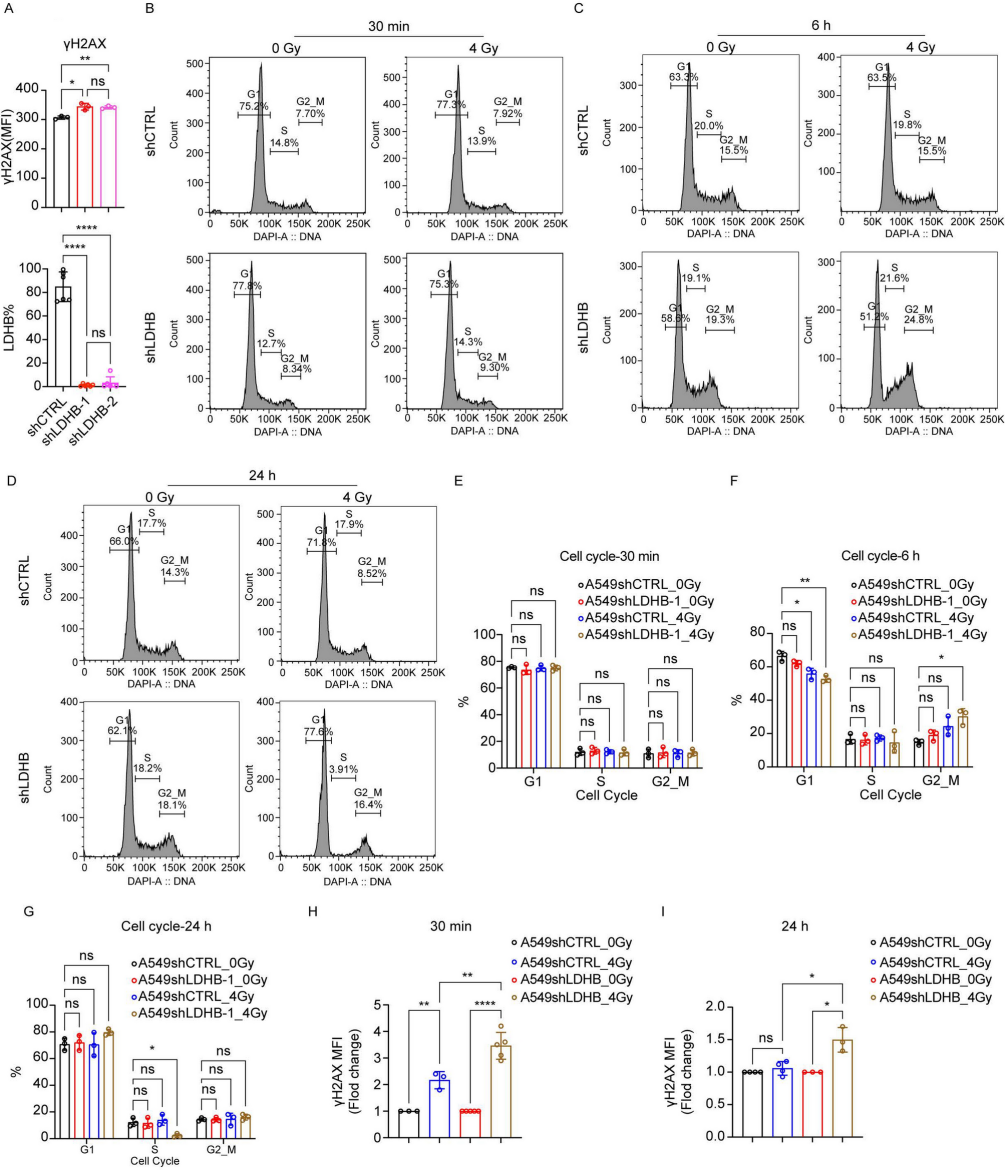
Long-term LDHB silencing renders cells sensitive to radiotherapy. **A**, the expression of LDHB and γH2AX in A549shCTRL and shLDHB cells was analyzed by flow cytometry (n = 3–6), and data were means ± SD, unpaired Welch’s 12 t-test or unpaired two-tailed t-test. **B**-**I**, Cell cycle, and DNA damage were analyzed with DAPI and γH2AX by flow 13 cytometry at the indicated time points after 4Gy irradiation (n = 3–4), data were means ± SD, unpaired Welch’s t-test**.** 14.

To avoid non-specific transfection effects^22,23^ and enable long-term LDHB silencing for in vivo studies, we established the stable LDHB silencing in A549 cells using small hairpin RNA (shLDHB, with two targeting sequences) or a scrambled control (shCTRL), as previously described^18,24^. In detail, we showed before that long-term LDHB silencing resulted in metabolic rewiring, e.g., decreased LDHA expression but increased NAD + and NADH levels^18^. In this study, our analysis on the single cell level by flow cytometry revealed that stable LDHB silencing was maintained after three passages (Fig. 2A, Fig. S1G). Approximately 10% of long-term LDHB silenced A549 cells featured increased H2AX phosphorylation compared to control (Fig. 2A, Fig. S1G). Indeed, the average H2AX phosphorylation measured of the total A549 population was significantly increased upon long-term LDHB silencing (Fig. 2A, right panel). Still, in contrast to acute LDHB silencing by siRNA transfection (Fig. 1F), long-term LDHB silencing did not result in an S-phase arrest (Fig. 2B-G). Also, irradiation of A549-shCTRL cells with 4 Gy did not result in an S-phase arrest, neither after 30 min nor after 6 or 24 h (Fig. 2B-G). However, irradiation of A549-shLDHB cells with 4 Gy resulted in a G2/M-phase arrest detectable 6 h after irradiation and a complete S-phase depletion 24 h after irradiation, respectively (Fig. 2B-G). In agreement with these findings, irradiation of A549-shLDHB cells with 4 Gy further increased H2AX phosphorylation after 30 min, and the relative levels remained increased 24 h after irradiation compared to irradiated control cells (Fig. 2H-I).

### LDHB silencing combined with radiotherapy promotes DNA damage and cellular senescence

**Fig.3:**
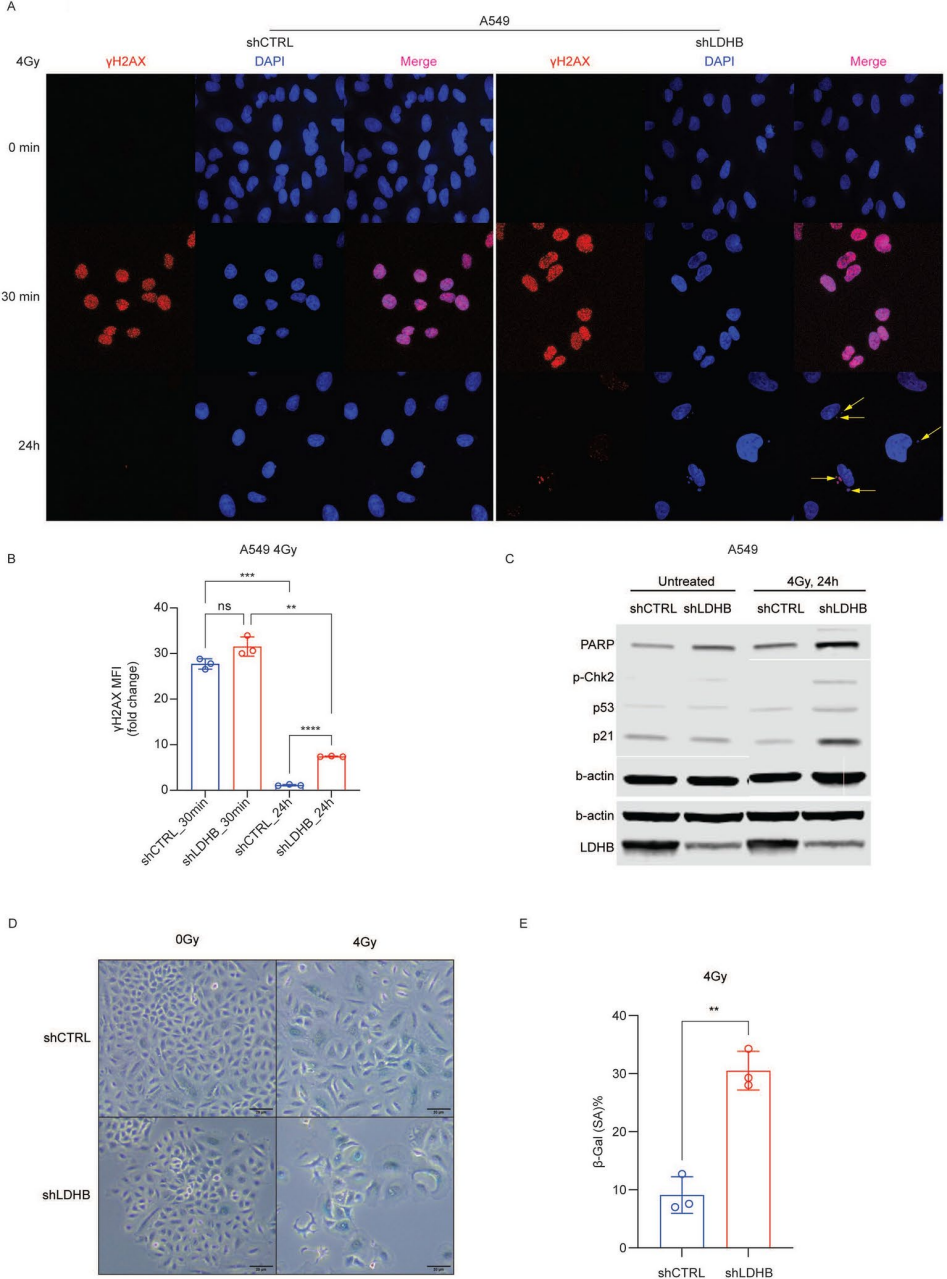
Long-term LDHB silencing combined with radiotherapy results in genomic instability. **A**-**B**, cells were stained with γH2AX and LDHB and imaged with confocal microscopy after treatment with 4Gy at different time points (n = 3), the 17 MFI was normalized to untreated shCTRL cells. data were means ± SD, unpaired Welch’s t-test. **C**, the expression of 18 PARP, p-Chk2, P53, P21, and LDHB was analyzed by western blot after 24 h of 4Gy irradiation. **D**-**E**, β-galactosidase 19 staining on shCTRL and shLDHB cells 5 days after irradiation with 4Gy (n = 3), data were means ± SD, unpaired Welch’s 20 t-test. 21.

Ionizing radiation induces DNA double-strand breaks, which can be detected by foci formation of phosphorylated histone H2AX (γH2AX)^20^. In A549 cells, γH2AX foci were detectable 30 min after exposure to 4 Gy, and the γH2AX signal intensity was further increased when radiotherapy was combined with LDHB silencing (Fig. 3B). In agreement, compared to both single treatments, combined LDHB silencing and RT resulted in increased protein levels of PARP, which promotes DNA repair at sites of DNA damage^25^. DNA double-strand breaks, if not repaired due to complex DNA modifications, deficiencies in the DDR machinery, or nucleotide shortage, can lead to the containment of broken fragments of DNA or whole chromosomes away from the rest of the genome in subcellular structures called micronuclei^26^. Only few micronuclei-containing cells were detectable 24 h after exposure of control cells to 4 Gy of ionizing radiation (Fig. 3A, left panels); however, a qualitative increase of micronuclei-containing cells was observed after combined LDHB silencing and radiotherapy exposure (Fig. 3A, right panels). The induction of DNA damage is associated with the activation of a cell cycle arrest^27^. Compared to single treatments, combined LDHB silencing and RT lead to increased p21 levels and β-Galactosidase staining, which are commonly used as a marker for cellular senescence, a state of permanent cell cycle arrest (Fig. 3C-E)^28^. Induction of senescence is associated with the accumulation of DNA double-strand breaks, as indicated by increased levels of γH2AX and phosphorylation of checkpoint kinase 2 (p-Chk2)^28^. Indeed, p-Chk2 levels were increased after combined LDHB silencing and exposure to RT (Fig. 3C).

### LDHB silencing depletes nucleotide metabolism in lung cancer tumors

**Fig.4:**
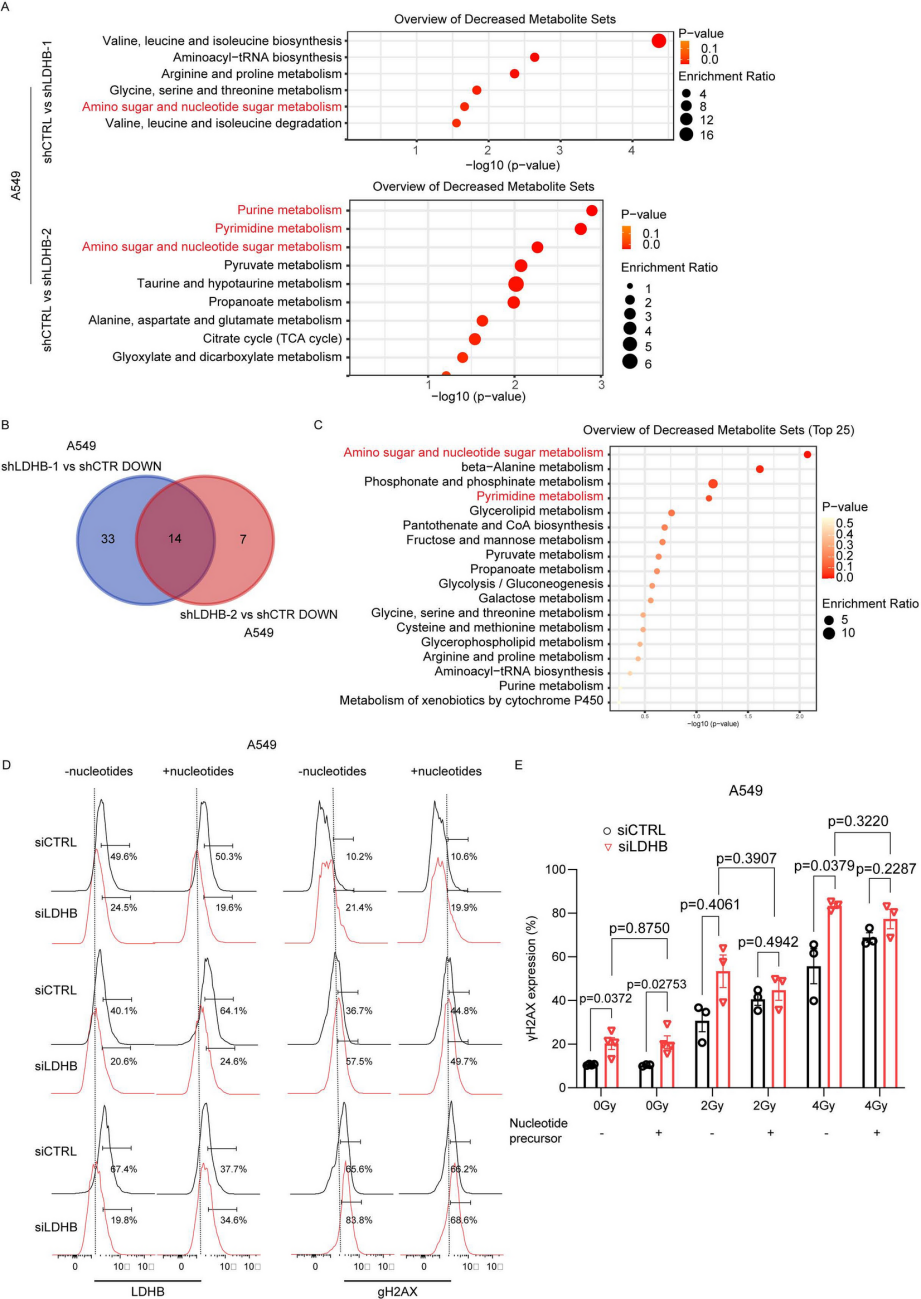
LDHB silencing depletes nucleotide metabolism in lung cancer tumors. **A**-**C**, Enrichment analysis of decreased metabolites in shLDHB tumors compared to shCTRL tumors. D-E, LDHB and γH2AX levels were analyzed in 23 A549 siCTRL and siLDHB cells after one hour of irradiation with or without supplementary with nucleotide precursors (100 24 μM hypoxanthine, 100 μM adenine, 400 μM uridine) (n = 3), data were means ± SD, unpaired Welch’s t-test. 25.

Upon induction of DNA damage, PARP activity triggers a metabolic shift critical for cell survival^25^. We showed before that short-term LDHB silencing in cell lines changes mitochondria-related metabolism, particularly nucleotide metabolism^18^. As a follow up, in the current study we characterized the metabolic changes induced by LDHB silencing in vivo, i.e., in A549 subcutaneous tumor xenografts described by us before^18^. In detail, our metabolite enrichment analysis revealed that the gene set “Amino sugar and nucleotide sugar metabolism” was among the most significantly depleted metabolite sets in both A549 shLDHB-1 and shLDHB-2 tumors compared to control (Fig. 4A). Intriguingly, the most decreased metabolite sets in A549 shLDHB-2 tumors compared to control was “Purine metabolism” and “Pyrimidine metabolism” (Fig. 4A). Consistently, A549 shLDHB-1 and shLDHB-2 tumors exhibited a shared decrease of 14 metabolites compared to control tumors (Fig. 4B), of which an enrichment analysis revealed that “Amino sugar and nucleotide sugar metabolism” and “Pyrimidine metabolism” were among the most significantly depleted metabolite sets (Fig. 4C).

As a proof-of-principle experiment to confirm that disruption of nucleotide metabolism underlies the phenotype induced by LDHB silencing, we tested whether nucleotide supplementation can protect cells from accumulation of DNA damage following LDHB silencing and RT exposure. In detail, supplementation with nucleotide precursors did not reduce the increased γH2AX levels upon LDHB silencing compared to controls (Fig. 4D and 4E, column #2 vs #4). Nucleotide supplementation further increased H2AX phosphorylation in control cells after exposure to 2 and 4 Gy of RT, though this increase was not statistically significant (Fig. 4E, column #5 vs #7 and column #9 vs #11, respectively). In contrast, the increased levels of H2AX phosphorylation upon combined LDHB silencing and exposure to either 2 or 4 Gy RT were actually reduced by supplementation with nucleotide precursors, though this decrease was also not statistically significant (Fig. 4E, column #6 vs #8 and column #10 vs #12, respectively). In summary, in vitro LDHB silencing in lung cancer cells decreases nucleotide metabolism, which is associated with increased DNA damage accumulation.

### LDHB silencing combined with radiotherapy leads to persistent DNA damage accumulation in lung cancer tumors

**Fig.5:**
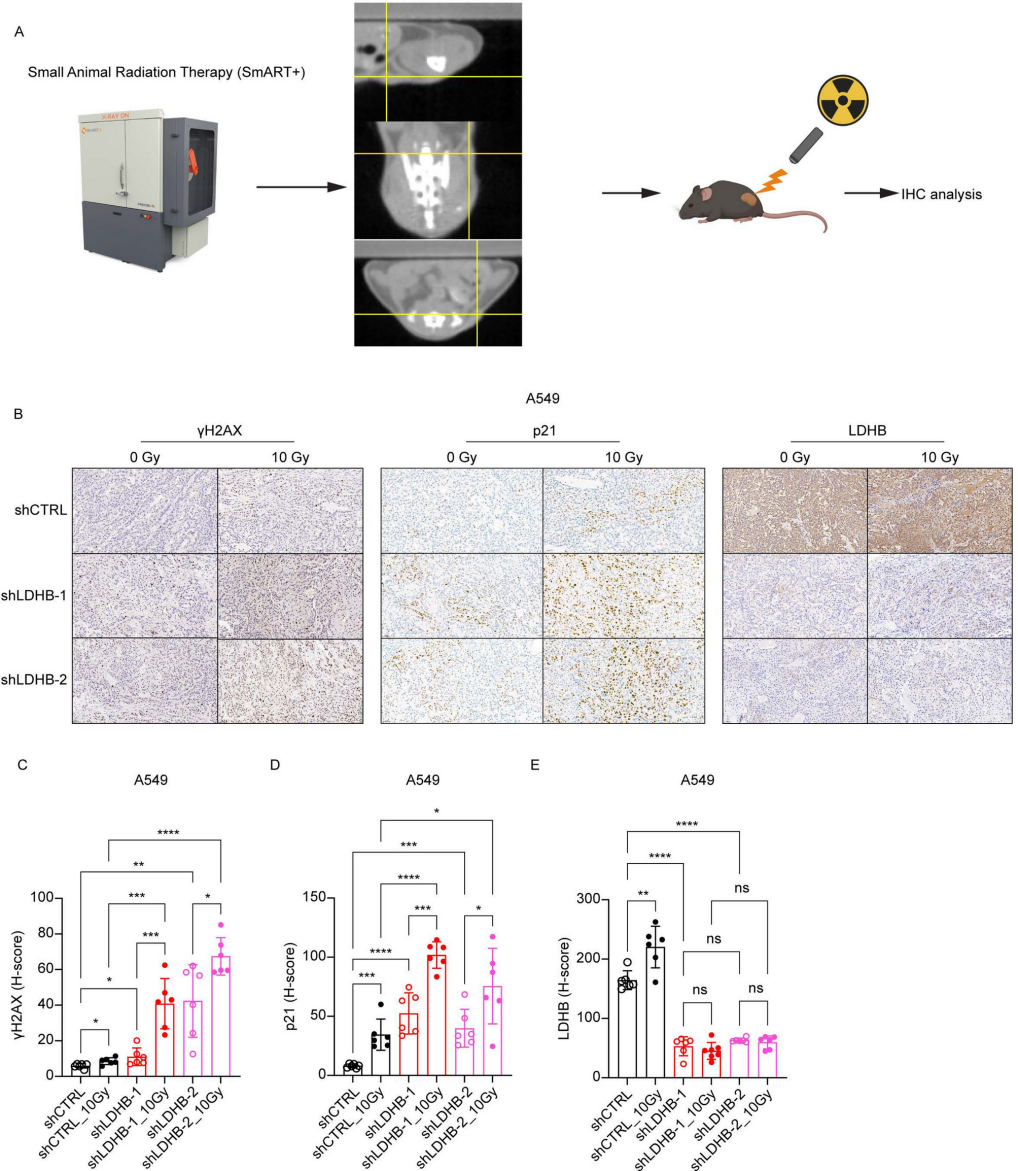
LDHB silencing depletes nucleotide metabolism in lung cancer tumors. **A**, schematic representation of local irradiation of xenograft tumors in mice. **B**-**E**, IHC analysis of LDHB, γH2AX and p21 expression in shCTRL and shLDHB 28 tumors 10 days after irradiation with 10Gy or control treatment (n = 6), data were means ± SD, two-tailed t-test. 29.

We showed before that LDHB silencing not only reduces the tumor initiation capacity but also the growth rate of A549 xenograft tumors compared to control tumors^18^. In a study currently under consideration for publication elsewhere, we showed that tumor growth of A549 xenografts is significantly delayed by LDHB silencing and completely blocked when LDHB silencing is combined with a single local irradiation with 10 Gy of ionizing radiation. At the end of the experiment, i.e., at least 20 days after RT exposure for all groups, γH2AX and p21 levels remained elevated in irradiated control tumors and in tumors with LDHB silencing alone, compared to unirradiated controls (Fig. 5B-E). Intriguingly, combining LDHB silencing with 10 Gy RT led to a dramatic increase in H2AX phosphorylation and p21 protein levels compared to either treatment alone (Fig. 5B-E). In addition, the LDHB expression was significantly increased in A549 shCTRL tumors after exposure to 10 Gy. Thus, the observed reduction of tumor growth after LDHB silencing reported by us before is associated with the accumulation of persistent DNA damage.

## Discussion

Two human patients were identified as entirely hereditary deficient for LDHB expression^29^. Further, a homozygous deletion of *LDHB* in the mouse model results only in a minor phenotype, e.g., increased lean body mass, and decreased total body fat/circulating insulin, but no immunodeficiency (www.mousephenotype.org, see also^18^). In cancer cells, the concentrations of the four dNTPs are, on average, 6 to 11 times higher compared to normal cells^30^. We previously demonstrated that LDHB silencing leads to imbalances in nucleotide pools^18^, which has been shown to result in DNA replication stress and subsequent DNA damage accumulation^31^. Intriguingly, the reanalysis of the TCGA data presented in this study revealed that LDHB expression in cancer cells positively correlated with a “Cell cycle score”, a “DNA damage score”, and a “DNA repair score” (Fig. 1A/B). Thus, we speculate that cancer cells require higher LDHB activity than normal cells to handle their increased nucleotide-related metabolic demands. Interestingly, LDHB silencing led to DNA damage, shown by increased H2AX phosphorylation, in both p53-wild type and p53-mutant cells (Fig. 1C/D). P53 coordinates nucleotide synthesis in response to DNA damage^32^ and acts as a master regulator of the cell cycle checkpoint machinery^27^. The p53 status determined the cell cycle phase where cancer cells arrested after LDHB silencing (Fig. 1F). It would be interesting to explore whether the p53 status after LDHB silencing influences the increased sensitivity of cancer cells to inhibitors specifically targeting the G1/S or G2/M checkpoints^27^.

Concerning radiotherapy, the role of LDHB expression appears to be complex. On the functional level, the double-knockout of LDHA and LDHB rendered murine melanoma and human colorectal adenocarcinoma cells more sensitive to RT^7^. In a study currently under consideration for publication elsewhere, we showed that long-term LDHB silencing in A549 cells further reduced survival after exposure to ionizing radiation. Addressing the underlying molecular mechanism, this study revealed that LDHB silencing combined with RT resulted in increased DNA damage accumulation in NSCLC cells compared to either treatment alone (Fig. 2H-J). Indeed, our in silico analysis and nucleotide supplementation experiments discussed below, suggest that LDHB silencing may deplete the nucleotide pools required for repairing DNA damage induced by RT. This is consistent with our previous studies in which pretreatment with pemetrexed, a nucleotide synthesis inhibitor, further augmented chemo- and radiotherapy-induced H2AX phosphorylation, resulting in increased anti-cancer efficiency of the combination regimens^33–35^.

Our in vivo experiments revealed that combined LDHB silencing and radiotherapy result in the accumulation of persistent DNA damage (Fig. 5). Interestingly, after combined LDHB silencing and exposure to radiotherapy in vitro, a significant fraction of cells contained micronuclei (Fig. 3A, right panels). Micronuclei are now recognized as mediators of the DNA damage response-associated immune recognition, which can alert the immune system to ongoing DNA damage, promoting immunological recognition and elimination of genetically unstable cells^26^. Indeed, we are currently investigating in an immune-competent lung tumor model^18,36^, whether LDHB silencing combined with radiotherapy initiates pro-inflammatory signaling cascades.

Our metabolite enrichment analysis revealed that A549 shLDHB-1 and shLDHB-2 tumors exhibited a shared decrease of 14 metabolites compared to control tumors (Fig. 4B) of which “Pyrimidine metabolism” was among the most significantly depleted metabolite sets (Fig. 4C). As expected, supplementation with nucleotide precursors reduced the increased levels of H2AX phosphorylation upon combined LDHB silencing and exposure to RT. However, nucleotide supplementation did not lower the increased γH2AX levels caused by LDHB silencing, and it actually increased H2AX phosphorylation in control cells when exposed to RT (Fig. 4E). Thus, we speculate that, after LDHB silencing, nucleotide supplementation brings the dNTP levels back within an optimal range for DNA repair. In contrast, supplementing control cells with additional nucleotides actually disturbs the optimal dNTP levels, resulting in inefficient DNA repair and thus increased γH2AX levels.

In summary, this study revealed that LDHB plays a role in maintaining metabolic balance, especially nucleotide metabolism, which is essential for optimal DNA repair activity in NSCLC cells. It further revealed that silencing LDHB in NSCLC cells enhances the effectiveness of radiotherapy to induce persistent DNA damage. The immune system plays a key role during RT for cancer^37^. Previous studies have shown that modulation of lactate metabolism, including directly targeting LDHB, has effects on immune cells^38,39^. Thus, it would be valuable to explore whether inhibition of LDHB can enhance the efficacy of RT in immunocompetent tumor models. Finally, it would be interesting to investigate if the immunostimulatory effects of persistent DNA damage caused by LDHB silencing in combination with RT can be further enhanced by additional immune checkpoint blockade.

## Supplementary Information

Supplementary Information 1.

Supplementary Information 2.

Supplementary Information 3.

## Data Availability

Availability of data and material All data generated or analyzed during this study are included in this published article and its supplementary information files. The single-cell RNA sequencing data was collected from Guillaumet-Adkins et al. (PDX_LUAD) (https://pubmed.ncbi.nlm.nih.gov/28249587/, https://www.ncbi.nlm.nih.gov/geo/query/acc.cgi?acc = GSE85534) and Lambrechts et al. (Lung) (https://pubmed.ncbi.nlm.nih.gov/29988129/, https://www.ebi.ac.uk/biostudies/arrayexpress/studies/E-MTAB-6149). The genes positively correlated with LDHB were generated by cBioPortal (https://www.cbioportal.org/) using the mRNA expression data from Cancer Cell Line Encyclopedia (https://pubmed.ncbi.nlm.nih.gov/31068700/, https://www.ncbi.nlm.nih.gov/sra?term = PRJNA523380).

## Abbreviations

NSCLC: Non-small cell lung cancer
RT: Radiotherapy
LDH: Actate dehydrogenase
LDHA: Lactate dehydrogenase A
LDHB: Llactate dehydrogenase B
*γ*H2AX: Phosphorylated histone H2AX
p-Chk2: Phosphorylation of checkpoint kinase 2
GSEA: Gene set enrichment analysis
