# Supplementary material for: LDHB silencing enhances the effects of radiotherapy by impairing nucleotide metabolism and promoting persistent DNA damage
Source: Sci Rep. 2025 Mar 29;15:10897. doi: 10.1038/s41598-025-95633-3 (PMC11954946; doi:10.1038/s41598-025-95633-3)
Supplement: Supplementary file 1 — Supplementary Information 1. [file 41598_2025_95633_MOESM1_ESM.pdf]

FIGURE LEGENDS

Fig.1. LDHB expression is linked to the activation of DNA damage response and its silencing increases DNA damage accumulation. *A-B, correlation analysis of LDHB expression with cell cycle and DNA repair score was performed using CancerSEA. C-E, the protein expression of LDHB and γH2AX was analyzed by flow cytometry 72 hours after transfection with siCTRL or siLDHB (n=3-4), and significance was evaluated using paired T-test. F, the cell cycle distribution was analyzed 72 hours after transfection with siCTRL or siLDHB by flow cytometry using DAPI staining. G, the γH2AX expression were analysis by flow cytometry at different cell cycle (n=3-4). data were means ± SD, unpaired Welch's t-test. H, LDHB expression in A549, H358, PF139-siCTRL and siLDHB cells was analyzed by Western blot after 72 hours of transfection.*

Fig.2. Long-term LDHB silencing renders cells sensitive to radiotherapy. *A, the expression of LDHB and γH2AX in A549shCTRL and shLDHB cells was analyzed by flow cytometry (n=3-6), and data were means ± SD, unpaired Welch's t-test or unpaired two-tailed t-test. B-I, Cell cycle, and DNA damage were analyzed with DAPI and γH2AX by flow cytometry at the indicated time points after 4Gy irradiation (n=3-4), data were means ± SD, unpaired Welch's t-test*.

Fig.3. Long-term LDHB silencing combined with radiotherapy results in genomic instability. *A-B, cells were stained with γH2AX and LDHB and imaged with confocal microscopy after treatment with 4Gy at different time points (n=3), the MFI was normalized to untreated shCTRL cells. data were means ± SD, unpaired Welch's t-test. C, the expression of PARP, p-Chk2, P53, P21, and LDHB was analyzed by western blot after 24 hours of 4Gy irradiation. D-E, β-galactosidase staining on shCTRL and shLDHB cells 5 days after irradiation with 4Gy (n=3), data were means ± SD, unpaired Welch's t-test.*

Fig.4. *LDHB silencing depletes nucleotide metabolism in lung cancer tumors*. *A-C, Enrichment analysis of decreased metabolites in shLDHB tumors compared to shCTRL tumors. D-E, LDHB and γH2AX levels were analyzed in A549 siCTRL and siLDHB cells after one hour of irradiation with or without supplementary with nucleotide precursors (100 μM hypoxanthine, 100 μM adenine, 400 μM uridine) (n=3), data were means ± SD, unpaired Welch's t-test.*

Fig.5. *LDHB silencing depletes nucleotide metabolism in lung cancer tumors*. *A, schematic representation of local irradiation of xenograft tumors in mice. B-E, IHC analysis of LDHB, γH2AX and p21 expression in shCTRL and shLDHB tumors 10 days after irradiation with 10Gy or control treatment (n=6), data were means ± SD, two-tailed t-test.*

Supplementary figure S1. *LDHB expression is linked to the activation of DNA damage response and its silencing increases DNA damage accumulation*. *A-D, the pathway enrichment analysis of LDHB-positive correlated genes in lung adenocarcinomas was performed with cBioPortal, Erichr, appyters and bokeh. E, the protein expression of LDHB was analyzed by flow cytometry 72 hours after transfection with siCTRL or siLDHB. F, the γH2AX expression were analysis by flow cytometry at different cell cycle. G, the expression of LDHB and γH2AX in A549shCTRL and shLDHB cells was analyzed by flow cytometry.*

Supplementary figure S2. *LDHB silencing depletes nucleotide metabolism in lung cancer tumors*. *A-B, the correlation analysis of the metabolites of A549shCTRL and shLDHB tumors was performed with MetabolAnalyst. C, the heatmap shows the top 50 dysregulated metabolites in A549 and shLDHB tumors. D, one-way ANOVA analysis of significantly dysregulated metabolites in A549shCTRL and shLDHB tumors. E-F, Enrichment analysis of upregulated metabolites in both shLDHB-1 and shLDHB-2 tumors compared to shCTRL tumors. G-H, metabolism map of dysregulated metabolites in shLDHB-1 and shLDHB-2 tumors compared to shCTRL tumors. n=6.*
