# Supplementary material for: LDHB silencing enhances the effects of radiotherapy by impairing nucleotide metabolism and promoting persistent DNA damage
Source: Sci Rep. 2025 Mar 29;15:10897. doi: 10.1038/s41598-025-95633-3 (PMC11954946; doi:10.1038/s41598-025-95633-3)
Supplement: Supplementary file 2 — Supplementary Information 2. [file 41598_2025_95633_MOESM2_ESM.pdf]

A549 shCTRL_0Gy_RGB

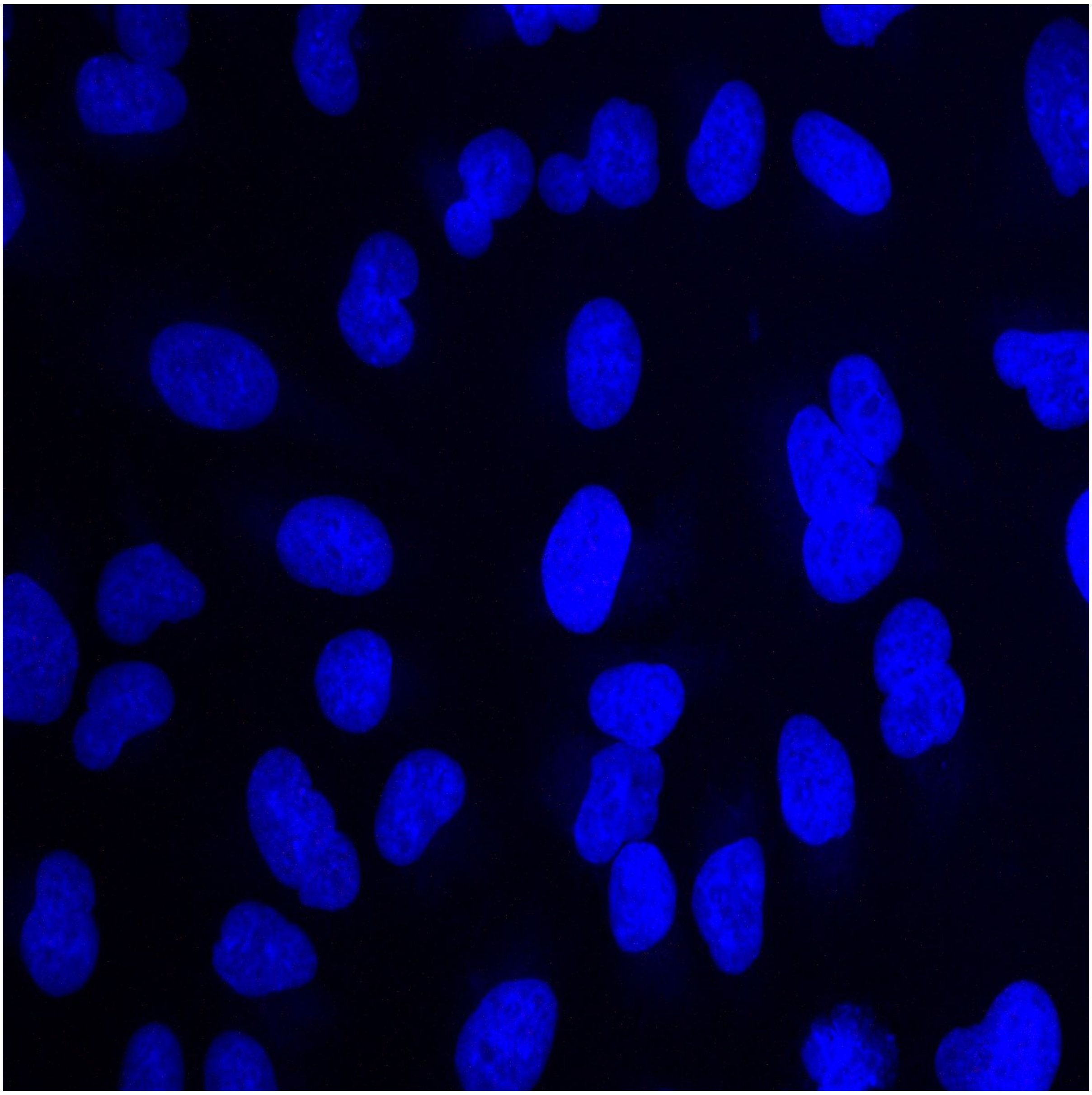

A549shCTRL 4Gy 30min_RGB

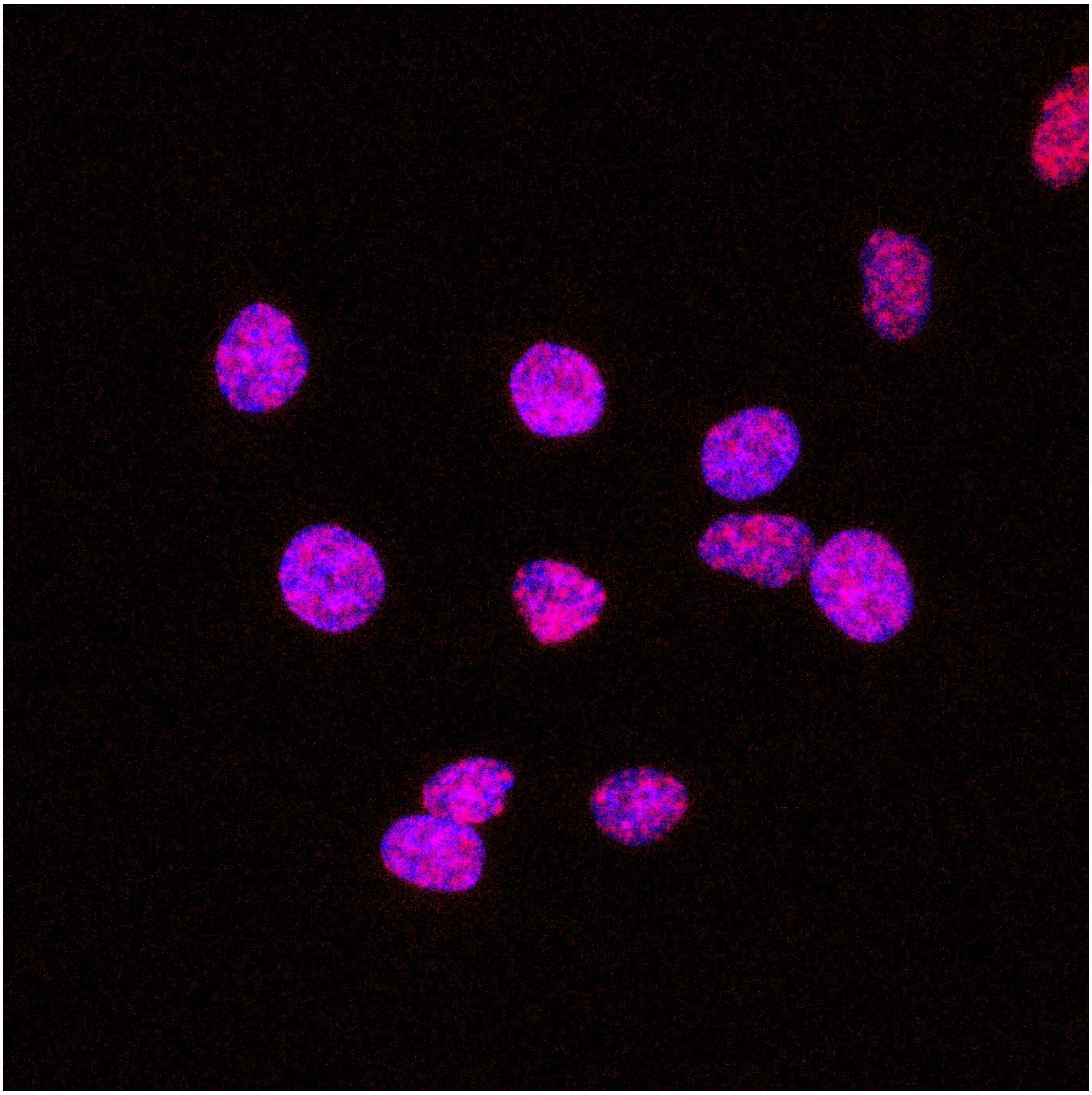

A549shCTRL 4Gy 24h_RGB

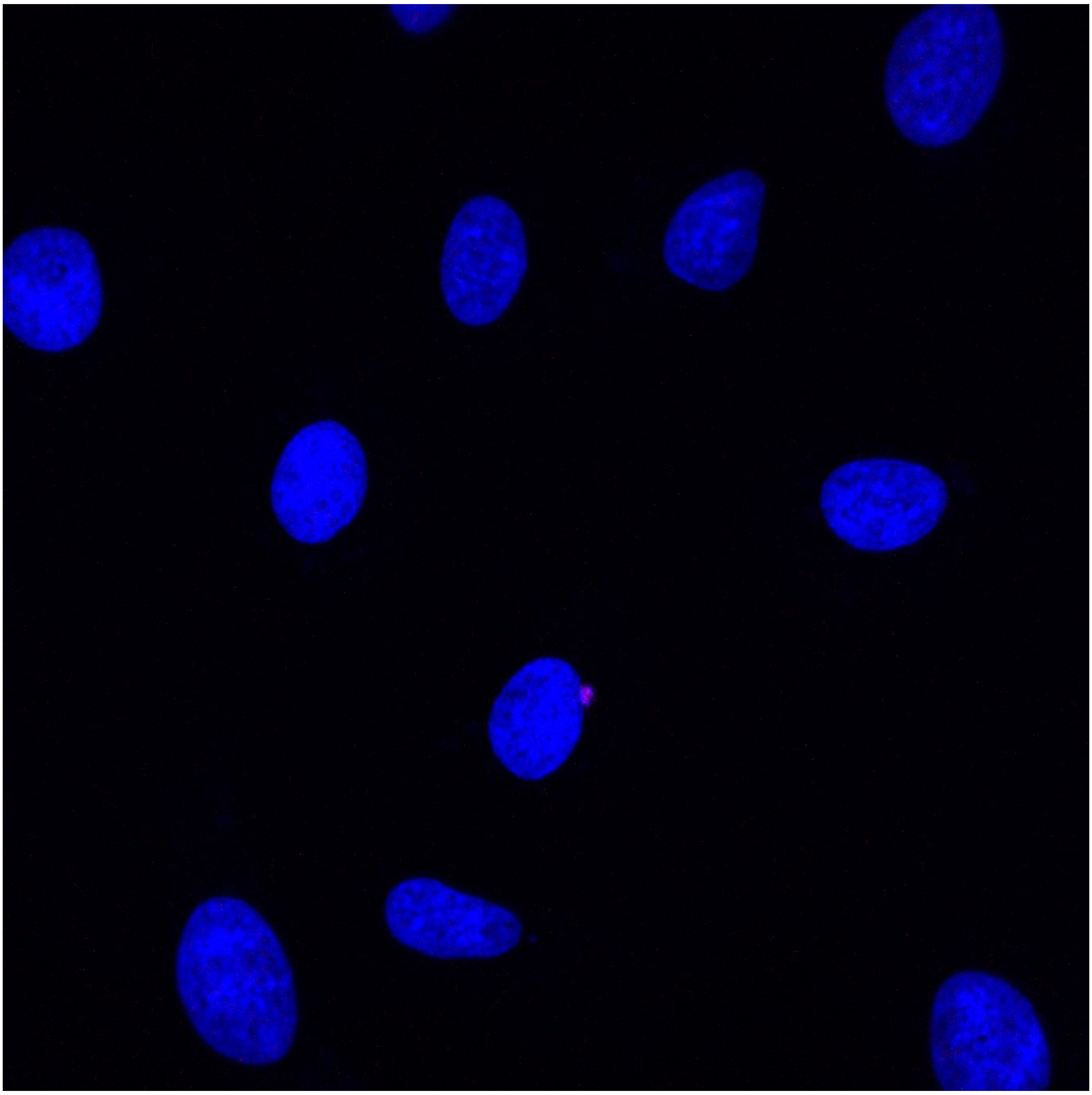

A549shLDHB_0Gy_RGB

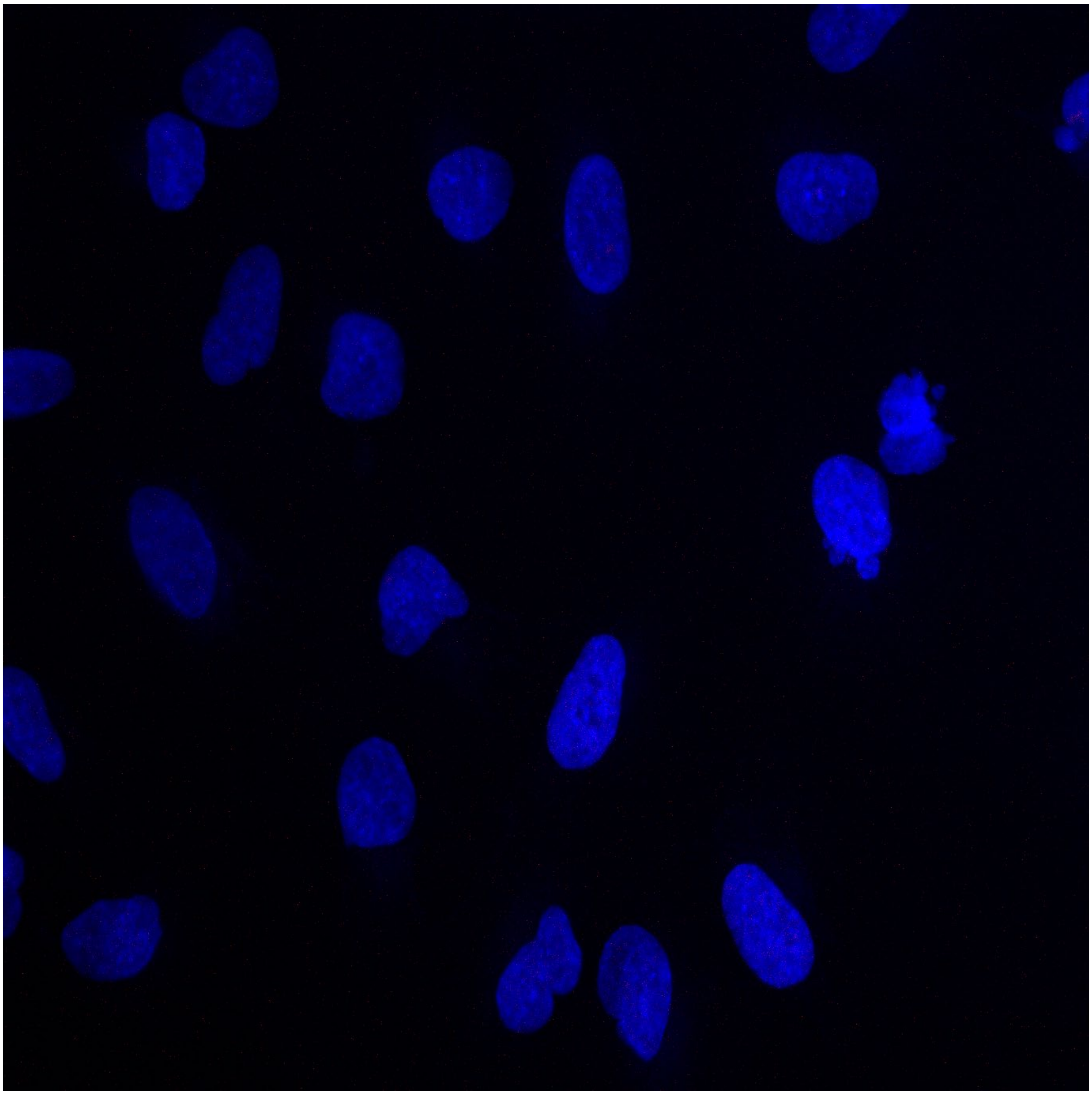

A549shLDHB_4Gy_30min_RGB

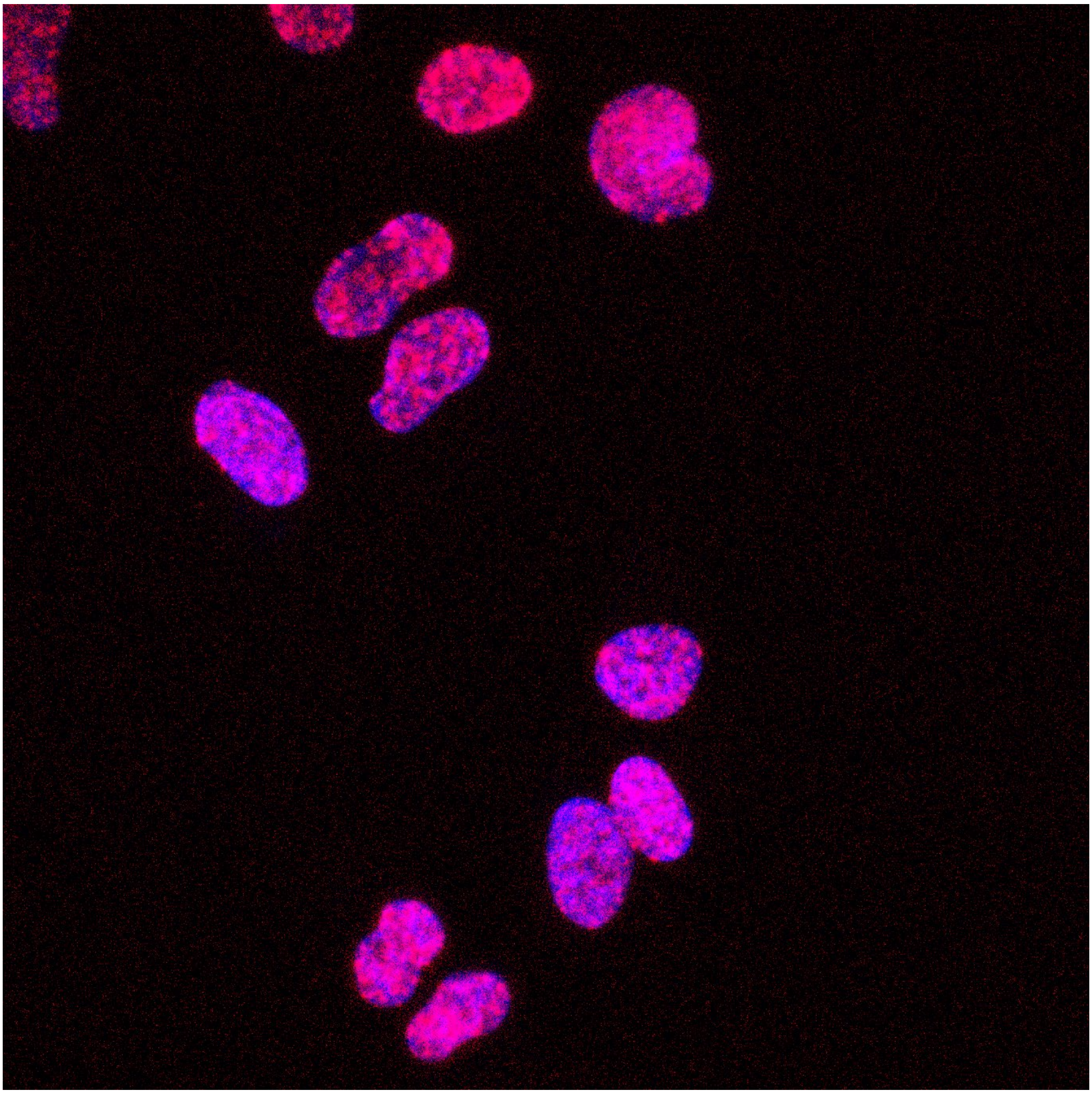

A549shLDHB_4Gy_24h_RGB

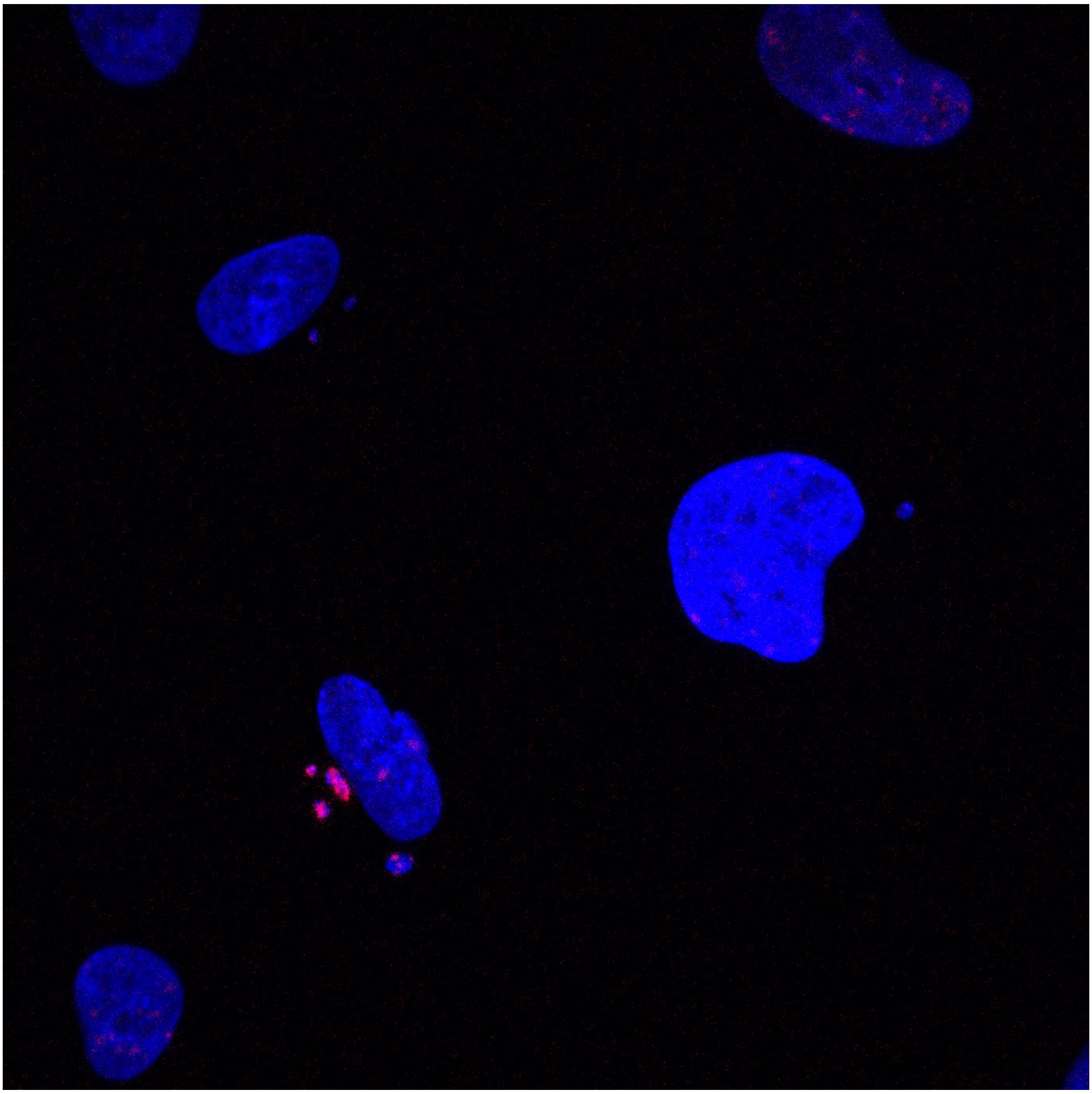

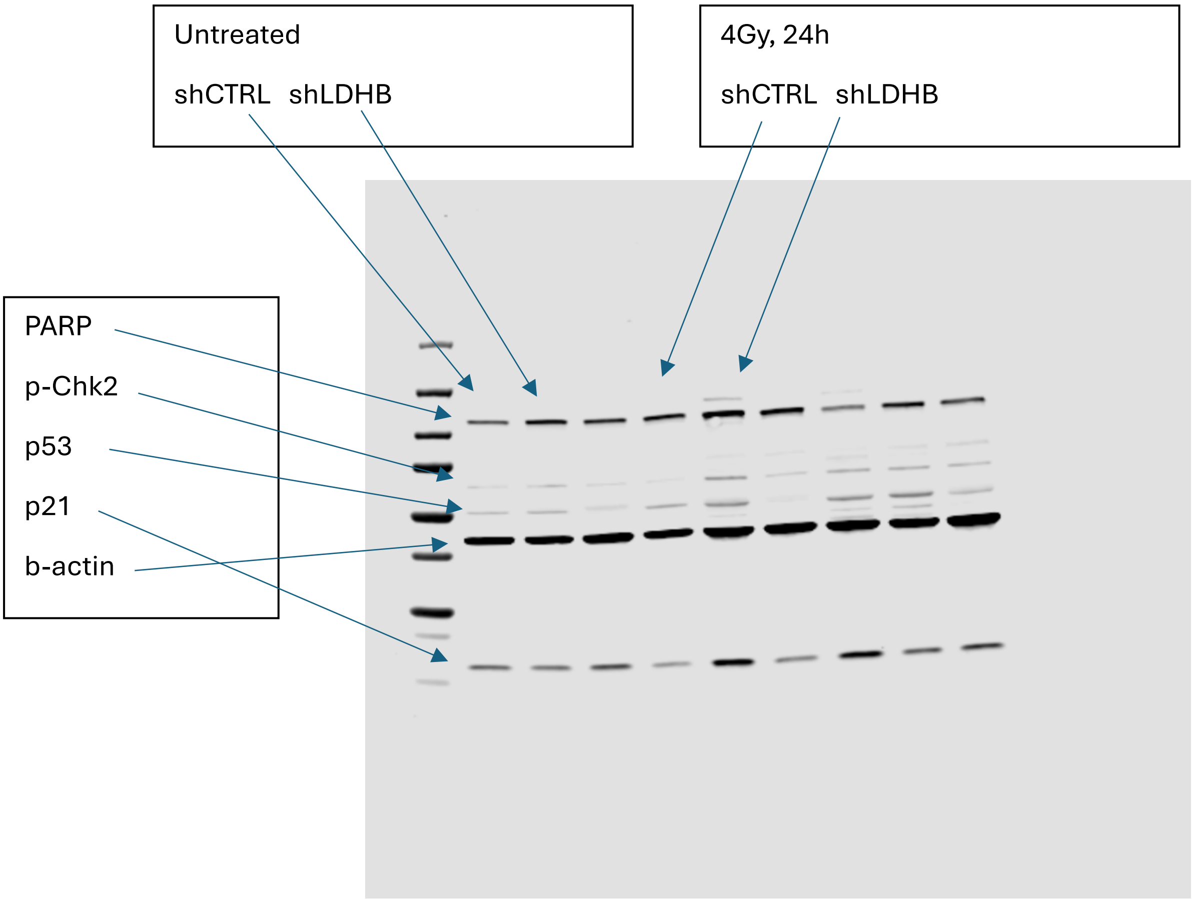
Untreated
shCTRL shLDHB
4Gy, 24h
shCTRL shLDHB
PARP
p-Chk2
p53
p21
b-actin
