# Supplementary figures and images for: LDHB silencing enhances the effects of radiotherapy by impairing nucleotide metabolism and promoting persistent DNA damage
Source: Sci Rep. 2025 Mar 29;15:10897. doi: 10.1038/s41598-025-95633-3 (PMC11954946; doi:10.1038/s41598-025-95633-3)

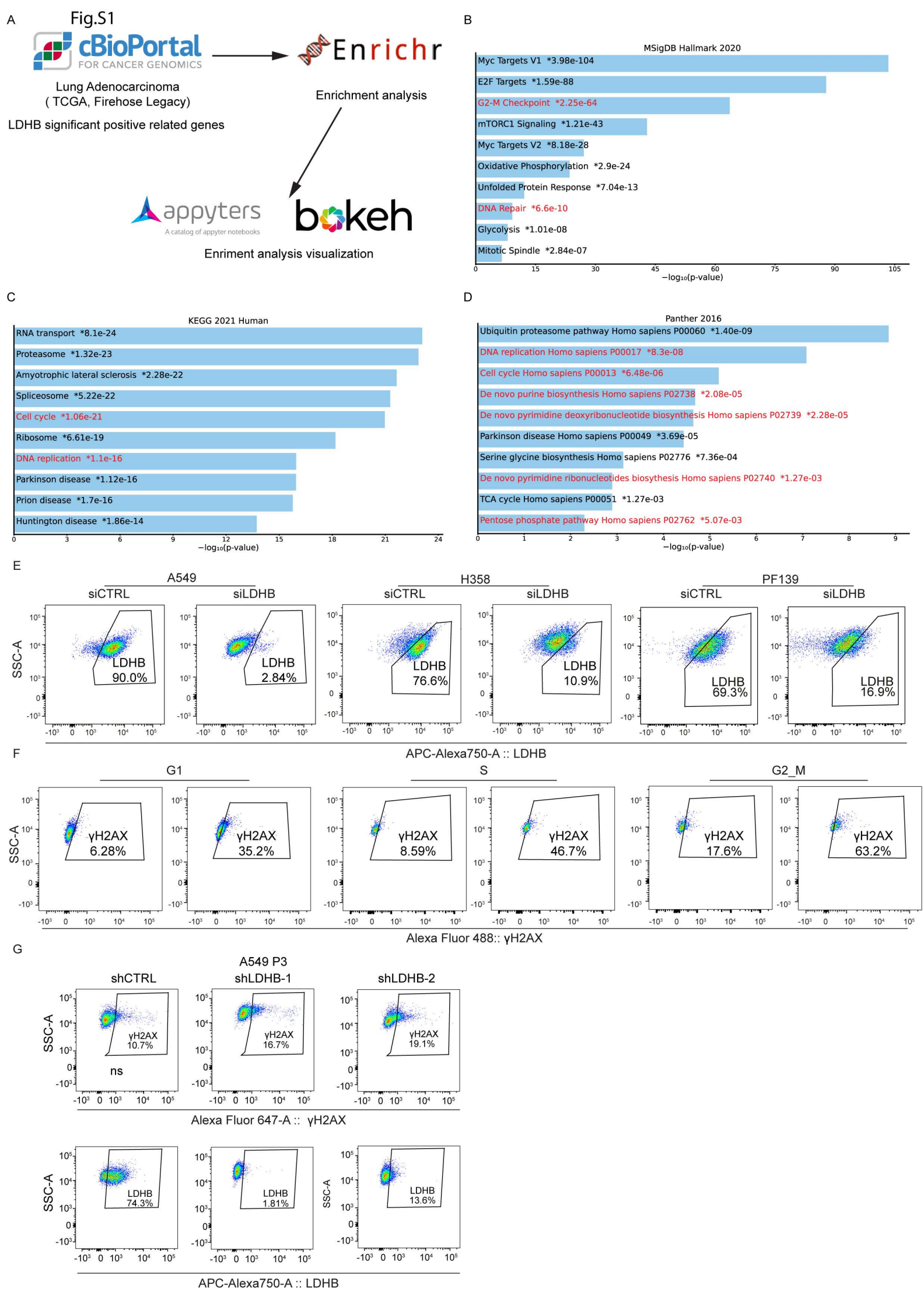

A

shCTRL vs shLDHB-1

Fig.S2

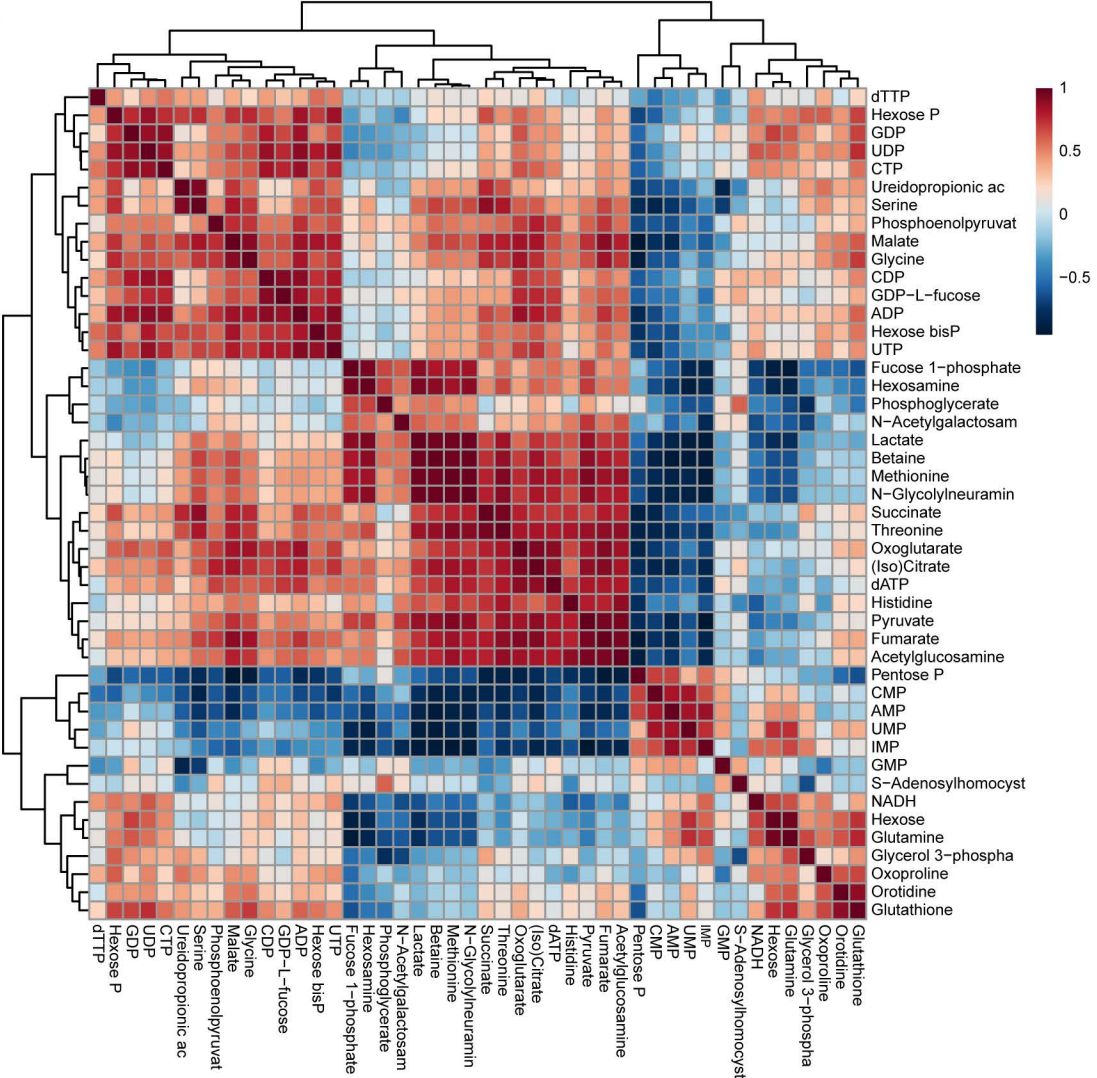

B

shCTRL vs shLDHB-2

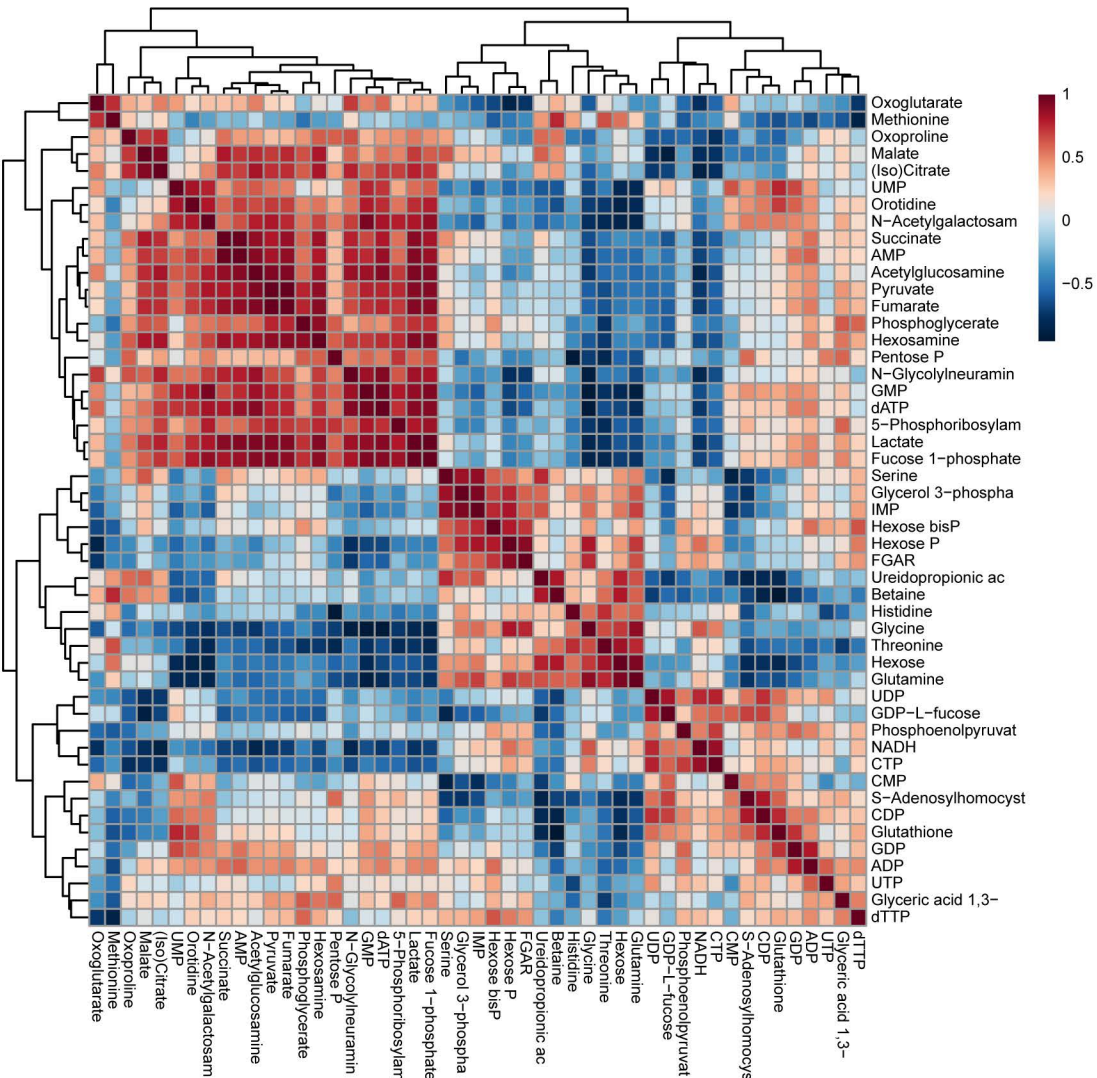

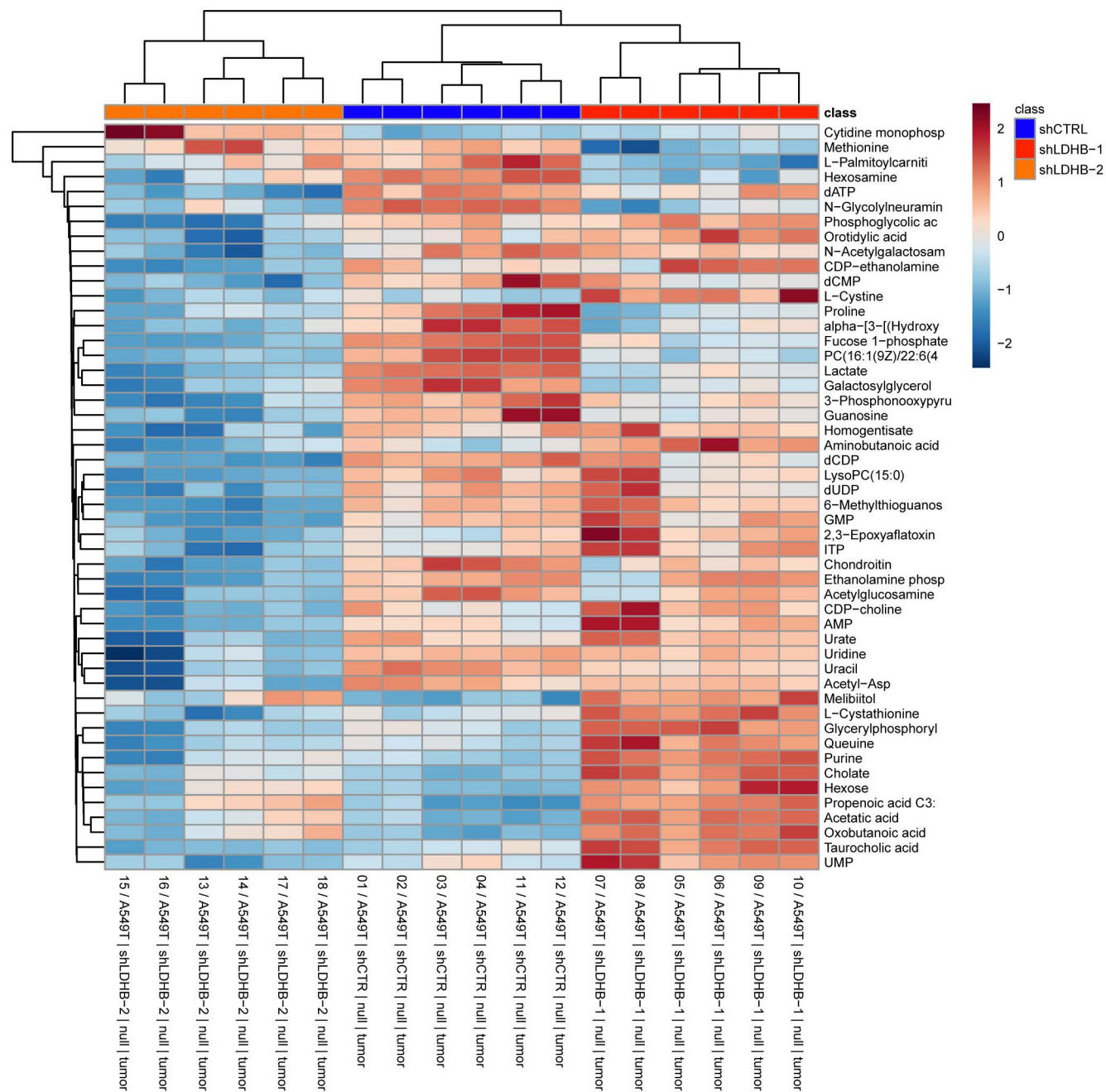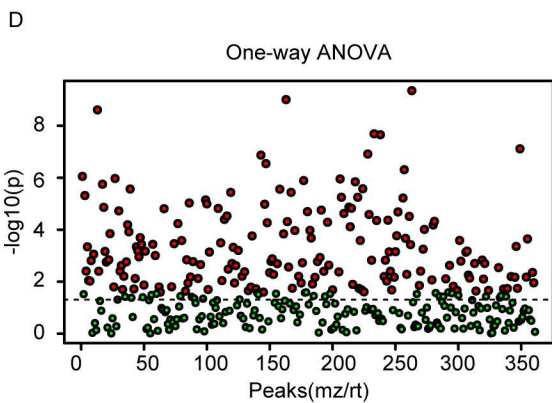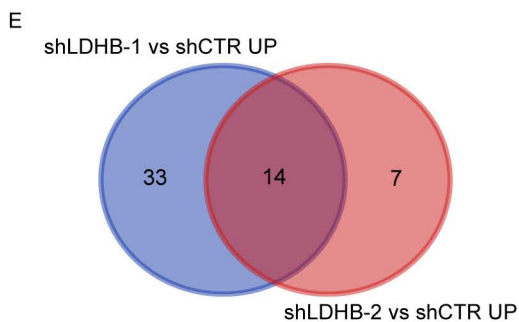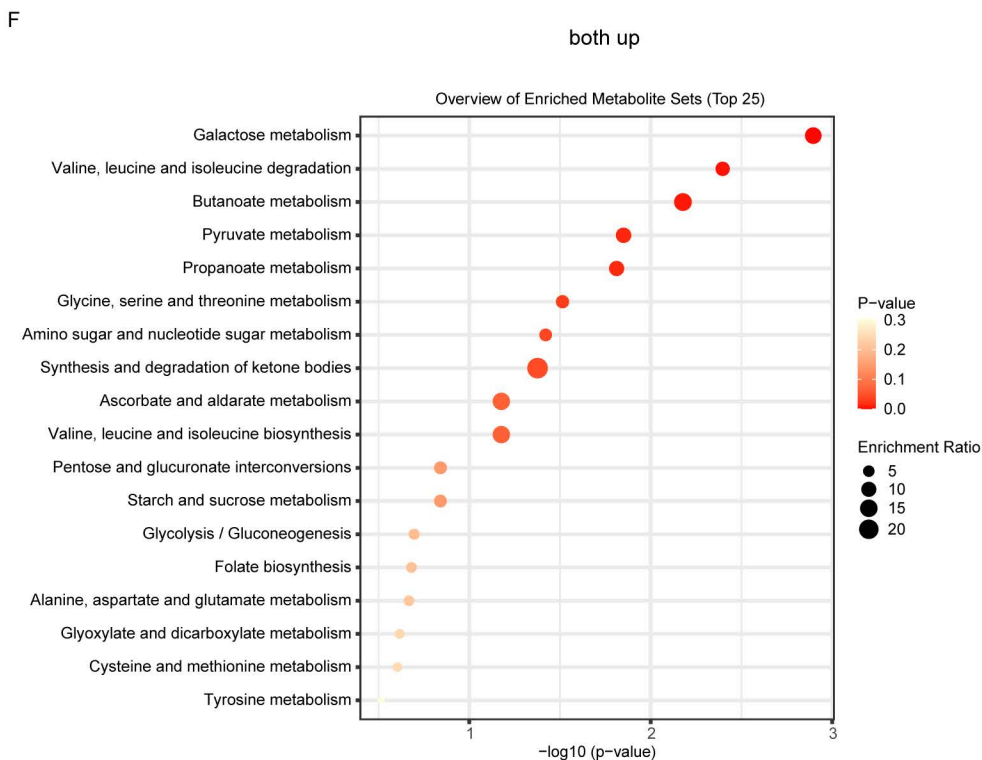

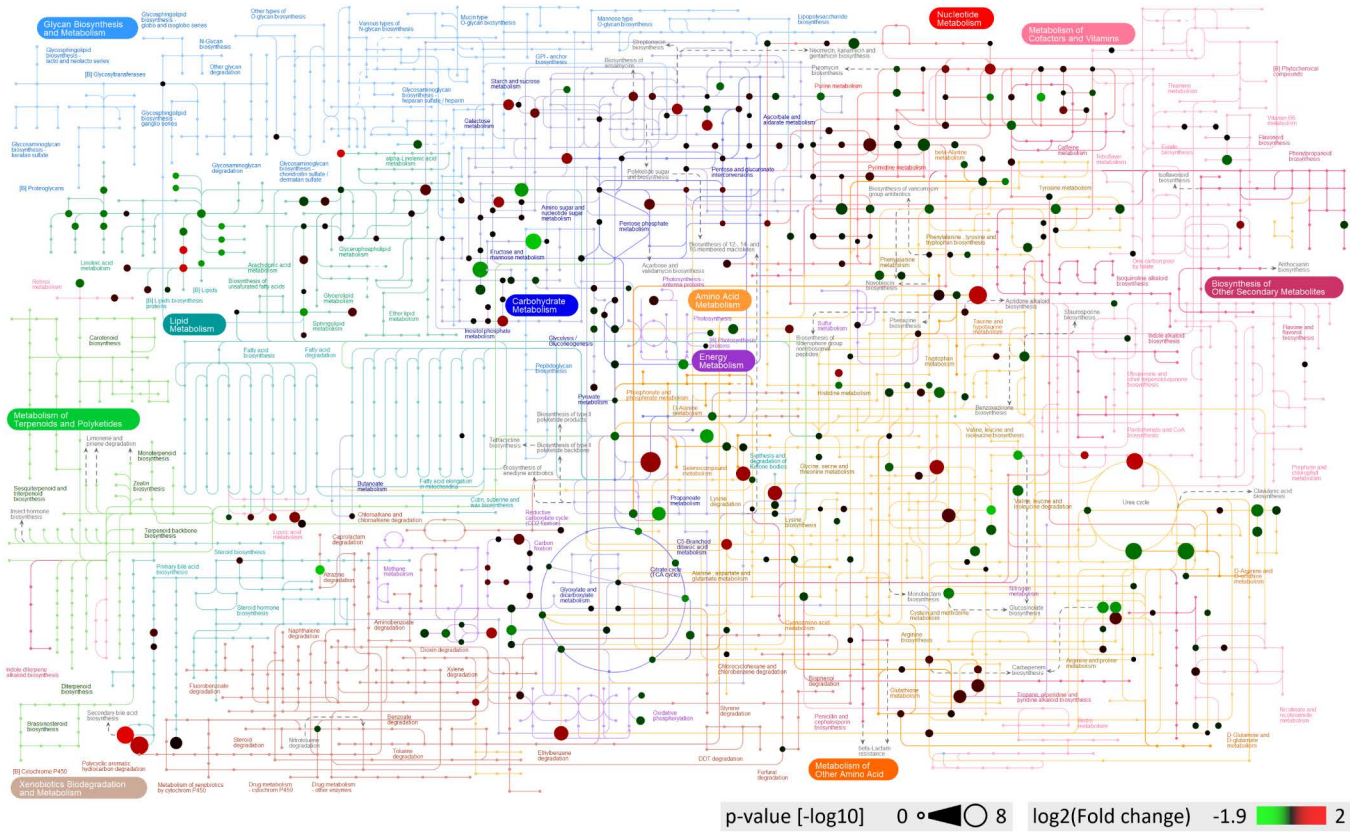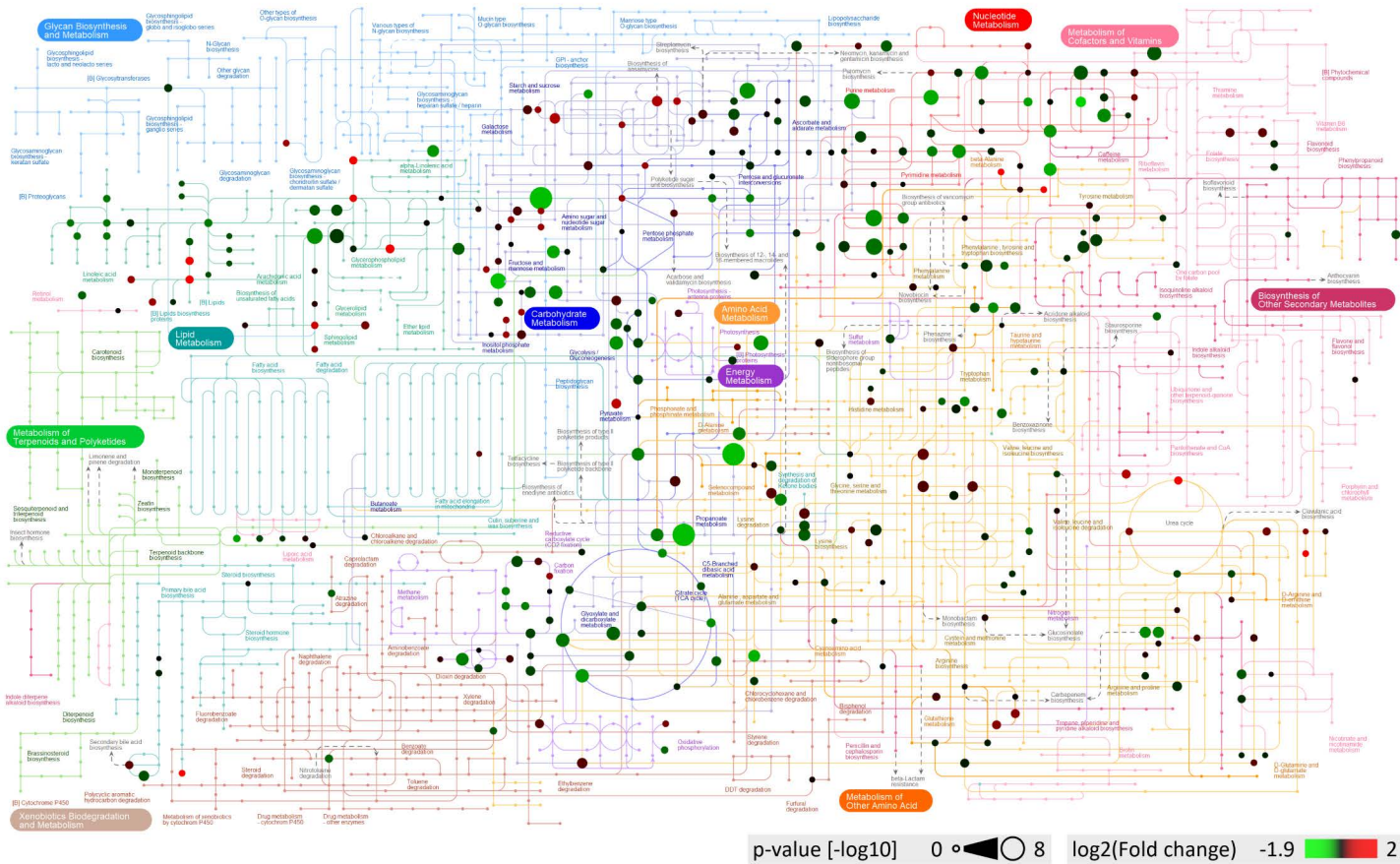

Supplement: Supplementary file 3 — Supplementary Information 3. [file 41598_2025_95633_MOESM3_ESM.pdf]
